# The importance of clinical experience in AI-assisted corneal diagnosis: verification using intentional AI misleading

**DOI:** 10.1038/s41598-025-85827-0

**Published:** 2025-01-09

**Authors:** Hiroki Maehara, Yuta Ueno, Takefumi Yamaguchi, Yoshiyuki Kitaguchi, Dai Miyazaki, Ryohei Nejima, Takenori Inomata, Naoko Kato, Tai-ichiro Chikama, Jun Ominato, Tatsuya Yunoki, Kinya Tsubota, Masahiro Oda, Manabu Suzutani, Tetsuju Sekiryu, Tetsuro Oshika

**Affiliations:** 1 Department of Ophthalmology, Fukushima Medical University School of Medicine, Fukushima, Japan; 2 Department of Ophthalmology, Faculty of Medicine, University of Tsukuba, 1-1-1 Tennoudai, Tsukuba, Ibaraki Japan; 3 Department of Ophthalmology, Tokyo Dental College Ichikawa General Hospital, Chiba, Japan; 4 Department of Ophthalmology, Osaka University Graduate School of Medicine, Osaka, Japan; 5 Division of Ophthalmology and Visual Science, Faculty of Medicine, Tottori University, Tottori, Japan; 6 Department of Ophthalmology, Miyata Eye Hospital, Miyazaki, Japan; 7 Department of Ophthalmology, Juntendo University Graduate School of Medicine, Tokyo, Japan; 8 Department of Ophthalmology, Tsukazaki Hospital, Hyogo, Japan; 9 Division of Ophthalmology and Visual Science, Graduate School of Biomedical and Health Sciences, Hiroshima University, Hiroshima, Japan; 10 Division of Ophthalmology and Visual Science, Graduate School of Medical and Dental Sciences, Niigata University, Niigata, Japan; 11 Department of Ophthalmology, University of Toyama, Toyama, Japan; 12 Department of Ophthalmology, Tokyo Medical University, Tokyo, Japan; 13 Graduate School of Informatics, Nagoya University, Nagoya, Japan; 14 Japan Anterior Segment Artificial Intelligence Research Group, Tsukuba, Japan

**Keywords:** AI, Artificial intelligence, Ocular surface, Misleading AI guidance, AI assist, Corneal diseases, Diagnosis

## Abstract

We developed an AI system capable of automatically classifying anterior eye images as either normal or indicative of corneal diseases. This study aims to investigate the influence of AI’s misleading guidance on ophthalmologists’ responses. This cross-sectional study included 30 cases each of infectious and immunological keratitis. Responses regarding the presence of infection were collected from 7 corneal specialists and 16 non-corneal-specialist ophthalmologists, first based on the images alone and then after presenting the AI’s classification results. The AI’s diagnoses were deliberately altered to present a correct classification in 70% of the cases and incorrect in 30%. The overall accuracy of the ophthalmologists did not significantly change after AI assistance was introduced [75.2 ± 8.1%, 75.9 ± 7.2%, respectively (*P* = 0.59)]. In cases where the AI presented incorrect diagnoses, the accuracy of corneal specialists before and after AI assistance was showing no significant change [60.3 ± 35.2% and 53.2 ± 30.9%, respectively (*P* = 0.11)]. In contrast, the accuracy for non-corneal specialists dropped significantly from 54.5 ± 27.8% to 31.6 ± 29.3% (*P* < 0.001), especially in cases where the AI presented incorrect options. Less experienced ophthalmologists were misled due to incorrect AI guidance, but corneal specialists were not. Even with the introduction of AI diagnostic support systems, the importance of ophthalmologist’s experience remains crucial.

**Supplementary Information:**

The online version contains supplementary material available at 10.1038/s41598-025-85827-0.

## Introduction

Blindness caused by keratitis represents the fifth leading cause of blindness globally^1–3^. Keratitis affects not only in the elderly populations but also in younger individuals, leading to potential lifelong vision loss^4,5^, which is regarded as “preventable blindness” because it does not cause severe visual impairment when diagnosed and treated properly at its early stages. As one of the current main limitations is medical assessment, artificial intelligence (AI)-based diagnosis is anticipated to be transformative in the management of keratitis. The integration of AI into medical diagnostics represents a significant advancement with the potential to improve diagnostic accuracy, streamline patient care, and facilitate early diseases detection^6–8^. AI is being used to assist in the diagnosis and management of various conditions, including infectious keratitis and other ophthalmic diseases^9–12^. We have developed a classification AI tool, called “CorneAI” that categorizes corneal conditions into nine disease states using a slit lamp microscope with diffuser light. Additionally, by combining it with a pathogen classification program for infectious diseases^10^, AI diagnostic support using CorneAI was developed and is being implemented in society^13^. CorneAI classifies various corneal conditions into nine categories: infectious keratitis, immunological keratitis, scarring, corneal deposition/dystrophy, bullous keratopathy, ocular surface tumors, cataract/IOL (intraocular lens) opacification, primary angle-closure glaucoma, and normal conditions. CorneAI utilizes YOLO V.5 as its analysis engine, with the area under the curve for normal eyes and corneal diseases ranging from 0.968 to 0.998. The support of CorneAI has improved the diagnostic accuracy of ophthalmologists^14^, suggesting the potential for AI to be utilized in clinical practice. The accuracy of CorneAI in a clinical setting was 86.0%^14^.

Although AI diagnostic performance for corneal diseases exceeds 0.99 of area under ROC curve, it does not achieve 100% accuracy^12^. Concerns remain regarding the reliability of these AI systems, particularly in instances where AI outputs misleading or incorrect diagnostic guidance. Such inaccuracies may significantly impact the decision-making process of medical professionals. This aspect of AI is crucial as the ultimate responsibility for patient diagnosis and care rests with the clinician, not the AI^15,16^. Resident physician, in particular, may be more susceptible to accepting AI-generated results without sufficient scrutiny, necessitating heightened vigilance. Even experienced ophthalmologists might struggle with the diagnosis of rare corneal diseases due to limited clinical experience^3,17^. We hypothesize that ophthalmologists could be misled by false diagnoses presented by AI. Therefore, our study aims to investigate how corneal specialists, board-certified ophthalmologists, and resident physician respond to incorrect diagnoses, infectious keratitis or immunological keratitis, presented by CorneAI.

## Materials and methods

The Institutional Review Board of the University of Tsukuba, Ibaraki, Japan, approved this prospective study protocol (ID: R3-108). The study adhered to the tenets of the Declaration of Helsinki, and all patients provided written informed consent after receiving a detailed explanation of the study protocols and possible consequences associated with participation. This study was multicenter collaborative prospective research. Slit-lamp images were collected from multiple collaborating facilities.

CorneAI was developed using 5,270 slit-lamp images collected from 18 institutions affiliated with the Japan Cornea Society. All images were meticulously verified by four corneal specialists, who validated diagnoses made by tertiary centers. They classified corneal diseases and cataracts into nine categories that encompass the major anterior segment diseases of the eye. We employed You Only Look Once (YOLO) Version 5 (YOLO V.5) as the AI algorithm to perform the nine-category classification. The model parameters in YOLO V.5 were pretrained using the Common Objects in Context dataset, followed by fine-tuning with the training dataset. YOLO V.5 was trained for 200 epochs with a mini-batch size of 16. The YOLO V.5 model achieved an area under the curve (AUC) ranging from 0.931 to 0.998, a sensitivity of 0.628 to 0.958, and a specificity of 0.969 to 0.998^14^.

**Fig. 1:**
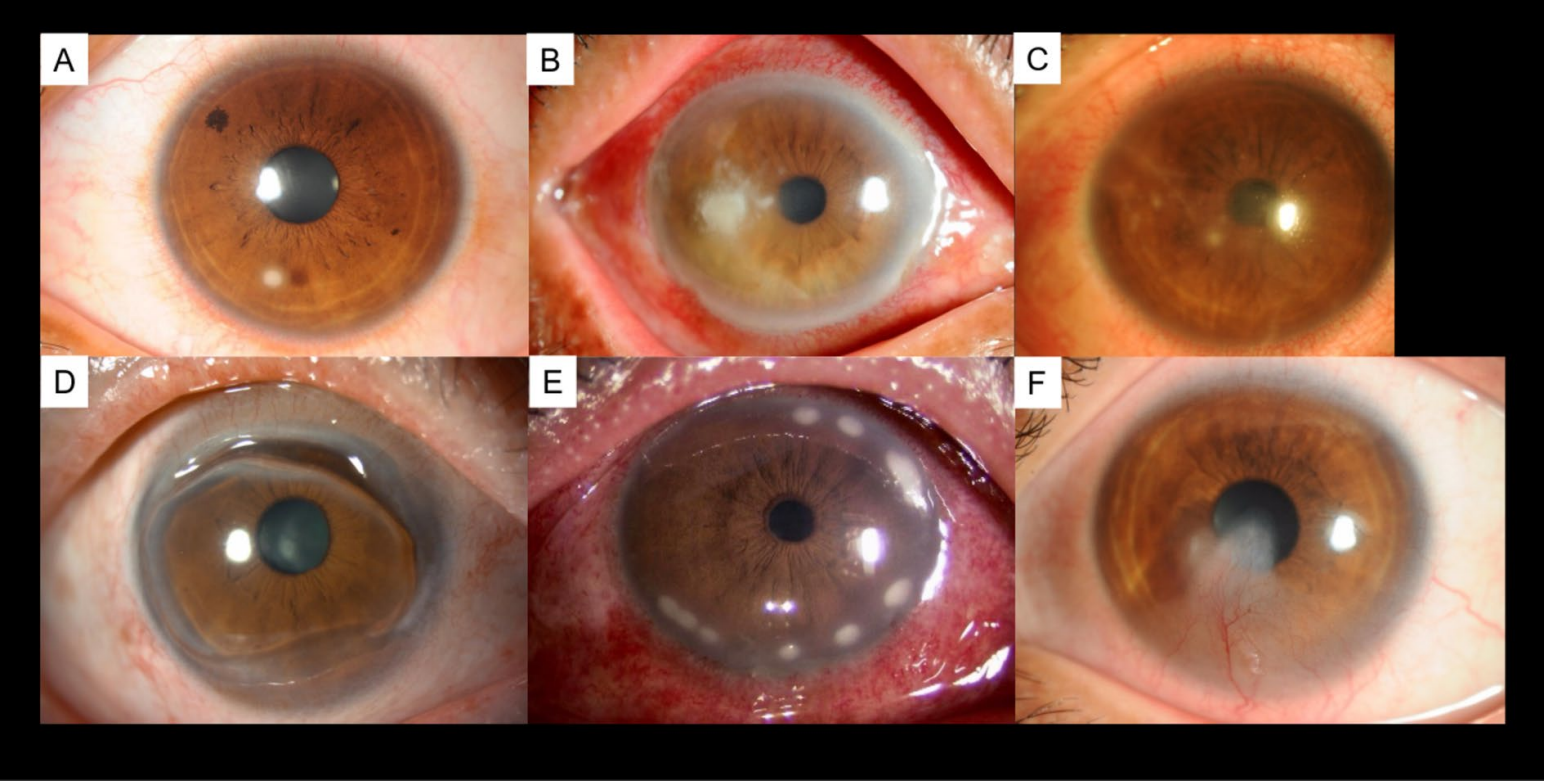
Representative cases each of disease. (**A**) Bacterial keratitis, (**B**) fungal keratitis, (**C**) acanthamoeba keratitis, (**D**) peripheral ulcerative keratitis, (**E**) marginal keratitis, and (**F**) phyctenular keratitis. Each disease was diagnosed by three or more corneal specialists, and for infectious keratitis, the culture results were also used in the diagnosis.

**Fig. 2:**
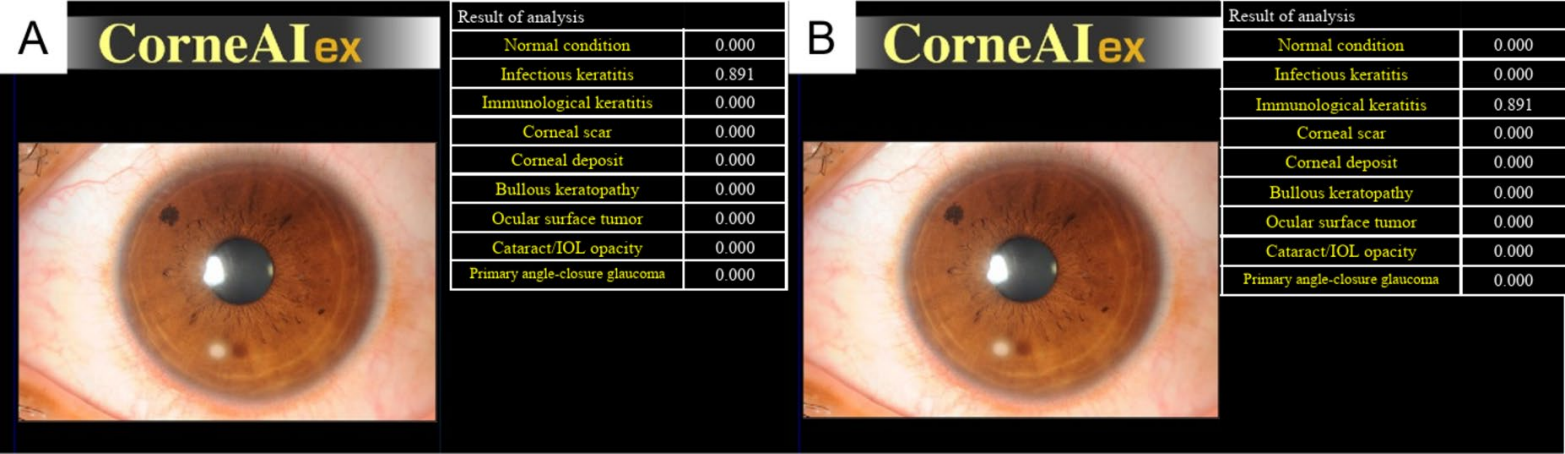
CorneAI’s discrimination results and edited images. (**A**) Discrimination results of anterior segment color photographs of patients with infectious keratitis identified by CorneAI. (**B**) The author edited the images from (**A**), resulting in images that show misleading AI outputs.

Slit-lamp images were obtained from multiple collaborating facilities. The slit-lamp images were taken at ×10 or ×16 magnifications in a darkroom, ensuring that at least the entire cornea was captured in a single image. This prospective study used anterior segment photographs registered in the Japan Ocular Imaging Registry^18^. Three corneal specialists (H.M., Y.U., and T.Y.) examined 680 cases. Among the cases with unanimous agreement in the diagnoses among the three experts, 60 cases were randomly selected (30 infectious keratitis and 30 immunological keratitis) as reported previously^13^. Infectious keratitis was defined to include bacterial keratitis, fungal keratitis, and acanthamoeba keratitis, while immunological keratitis encompassed peripheral ulcerative keratitis, marginal keratitis, and phyctenular keratitis, with 10 cases selected for each disease. Representative cases of each disease are shown in Fig. 1. In the dataset of 60 images in this study, CorneAI indicated a classification accuracy of 100% for both infectious keratitis and immunological keratitis. To simulate diagnostic challenges, the authors modified the diagnostic outputs of CorneAI to achieve a correct classification rate of 70% and an incorrect classification rate of 30% for each disease category (misleading AI outputs, Fig. 2). The misleading AI outputs were created by H.M., Y.U., and T.Y., who arbitrarily selected cases where the diagnosis between infectious keratitis and immunological keratitis was challenging and intentionally generated these images.

Twenty-three ophthalmologists participated in this prospective study. Seven corneal specialists, 7 board-certified specialists, and 9 residents were asked to classify a total of 120 images into infectious keratitis or immunological keratitis, with and without AI support (Fig. S1). Corneal specialists also hold board certification as ophthalmologists. Residents had less than 4 years of ophthalmology experience and were not board certified. In the classification with CorneAI support, CorneAI provided 9 classifications with likelihood values for each image (Fig. 2A).

First, the ophthalmologists were tasked with determining whether each of the 60 original images depicted infectious keratitis or immunological keratitis (First test). Subsequently, the same images, including misleading AI outputs, were presented to the ophthalmologists along with the interpretation results from CorneAI for their assessment (Second test). We compared the performance of classification between with and without AI support, and among corneal specialists, board-certified specialists, and residents. Three corneal specialists (H.M., Y.U., and T.Y.) were excluded from the survey because they had prior knowledge of the correct diagnoses.

### Statistics

Statistical analyses were conducted using JMP16 software (SAS Institute, Cary, NC, USA). The Wilcoxon rank sum test was used to compare the accuracy of diagnosis and the time for CorneAI and 23 ophthalmologists to complete classification of 120 images with and without AI support. Tukey-Kramer’s HSD test was used for comparison among the three groups. We also compared the results between corneal specialists, board-certified specialists and residents. The sample size was determined using a “Sample Size Calculator” with an alpha of 5% and a power (1 − β) of 0.80, yielding a required sample size of 6 participants. Cases with any missing clinical data were excluded from the analysis in this study. Likewise, a student’s t-test was performed for sensitivity analysis, and similar results were obtained. *P* < 0.05 was considered statistically significant.

## Results

Table 1 presents the years of experience and specialties of the responding ophthalmologists. A significant difference in years of ophthalmology experience was observed between corneal specialists and residents or board-certified ophthalmologists and residents (*P* = 0.0016 and *P* = 0.0011, respectively). However, no significant differences and found between corneal specialists and board-certified ophthalmologists (*P* = 0.068).

**Table 1:** Examiners profile.

		Corneal specialists	Board-certified ophthalmologists (non-corneal specialists)	Residents
N		7	7	9
Years in ophthalmology (Year)		14.7 ± 10.1	7.4 ± 2.5	2.9 ± 0.61
Specialties in ophthalmology	Cornea	7	0	0
	Glaucoma	0	2	0
	Ocular oncology	0	2	0
	Cataract	0	2	0
	None	0	1	9

Corneal specialists also hold board-certified as ophthalmologists.

**Fig. 3:**
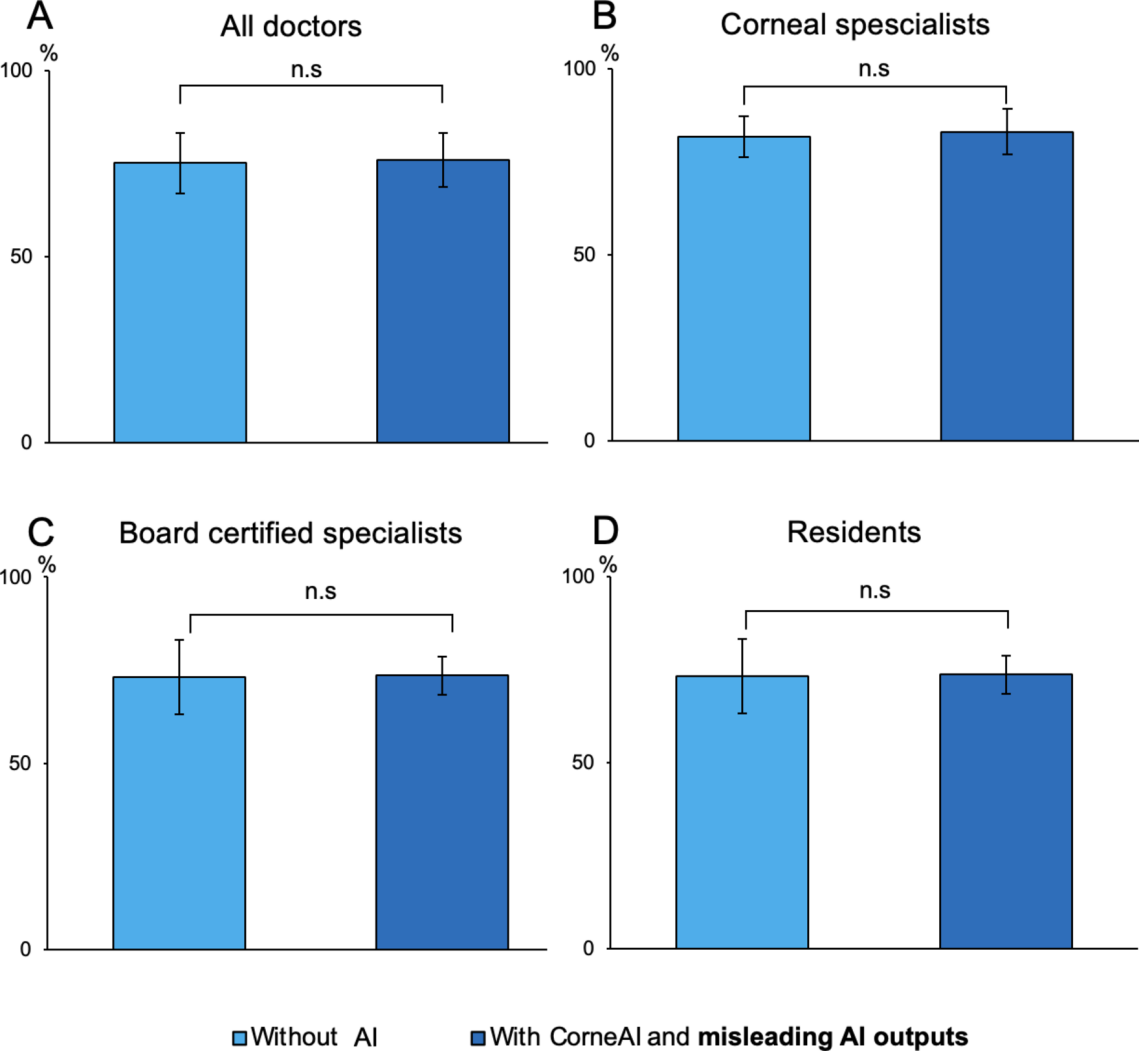
Change in ophthalmologists’ accuracy before and after CorneAI assistance, including misleading AI outputs. (**A**) All doctors. (**B**) Corneal specialists. (**C**) Board certified specialists. (**D**) Residents. Because of inclusion of misleading AI outputs resulted in no change in the accuracy rates of ophthalmologists before and after AI assistance.

When considering all AI assistance, including misleading AI outputs, the overall accuracy rates of ophthalmologists’ diagnoses, before and after AI assistance, remained statistically unchanged across all questions (75.2 ± 8.1% before AI assistance, 75.9 ± 7.2% after AI assistance, *P* = 0.59). The accuracy rate for diagnosing infectious keratitis without AI assistance was 78.8 ± 27.7% among all ophthalmologists. With the inclusion of misleading AI outputs, the accuracy rate for diagnosing infectious keratitis using CorneAI was 80.3 ± 29.7%, with no significant difference before and after AI assistance (*P* = 0.63). Similarly, for immunological keratitis, the accuracy rates for all ophthalmologists were 71.5 ± 25.8% before AI assistance and 71.6 ± 32.4% after AI assistance, with no significant difference (*P* = 0.99). Changes in accuracy rates for corneal specialists, board-certified ophthalmologists, and residents for all questions, including those with misleading AI outputs, are shown in Fig. 3.

**Fig. 4:**
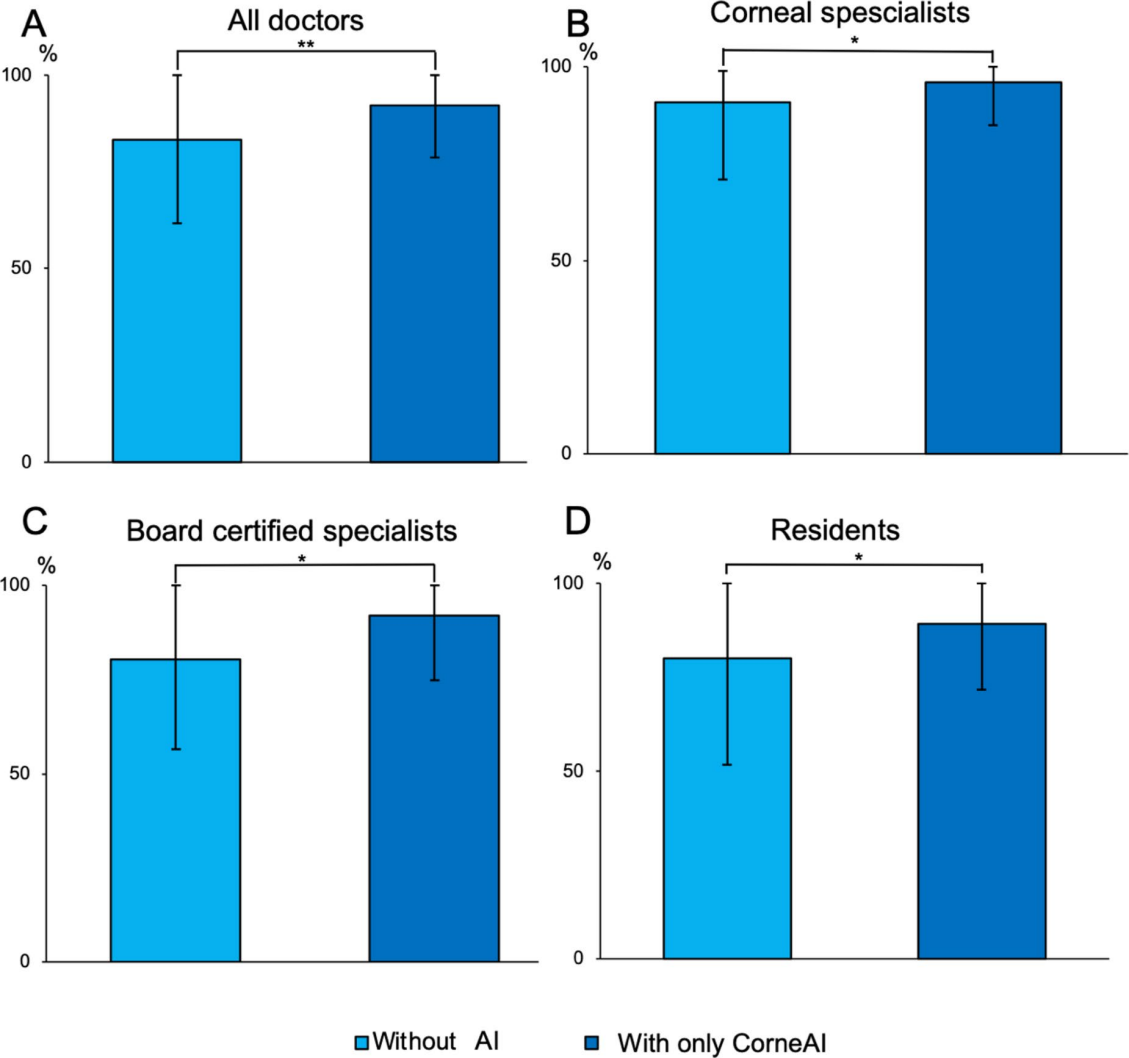
Change in ophthalmologists’ accuracy before and after AI assistance, only correct AI images. (**A**) All doctors. (**B**) Corneal specialists. (**C**) Board certified specialists. (**D**) Residents. (*P < 0.05, **P < 0.001). With correct answers provided by CorneAI support, there was a significant increase in the accuracy rates of ophthalmologists before and after AI assistance.

Of note, when assisted only by CorneAI, the overall accuracy rates of ophthalmologists significantly improved when CorneAI provided correct answers (83.3 ± 21.6% before AI assistance, 92.1 ± 13.4% after AI assistance, *P* < 0.001). Changes in accuracy rates for corneal specialists, board-certified ophthalmologists, and residents when CorneAI provided correct answers are shown in Fig. 4.

**Fig. 5:**
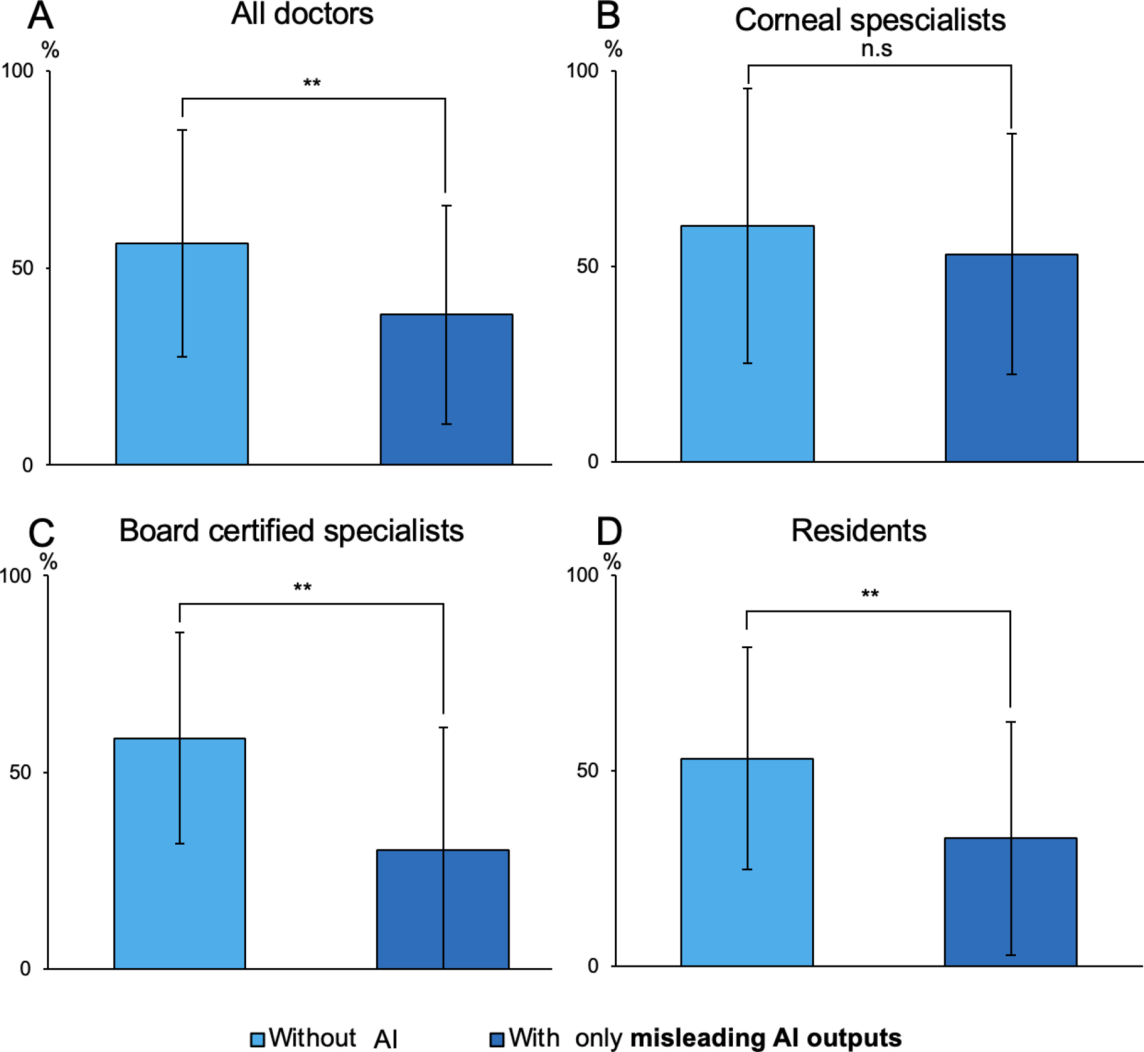
Change in ophthalmologists’ accuracy before and after AI assistance, only misleading AI outputs. (**A**) All doctors. (**B**) Corneal specialists. (**C**) Board certified specialists. (**D**) Residents. (**P < 0.001). All doctors, including board-certified specialists and residents, experienced a significant decrease in accuracy rates and made incorrect selections due to the presence of misleading AI outputs. However, Corneal specialists showed no change in accuracy rates even when presented with misleading AI outputs, indicating that they were not misled by the misleading AI outputs.

When ophthalmologists were assisted solely by misleading AI outputs, their overall diagnostic accuracy rates demonstrated a significant decline, decreasing from 56.3 ± 28.7% before AI assistance to 38.2 ± 27.8% after AI assistance (*P* < 0.001). The accuracy rate for board-certified ophthalmologists significantly decreased from 58.7 ± 26.8% to 30.2 ± 31.3% (*P* < 0.001), and for residents, it significantly decreased from 53.1 ± 28.4% to 32.7 ± 29.9% (*P* < 0.001). However, there was no significant difference in the accuracy rates for corneal specialists when referencing misleading AI outputs (60.3 ± 35.2% before AI assistance, 53.2 ± 30.9% after AI assistance, *P* = 0.11) (Fig. 5). Additionally, in the context of misleading AI outputs, corneal specialists demonstrated significantly higher accuracy rates compared to board-certified ophthalmologists and residents (*P* = 0.039).

The accuracy rates for all ophthalmologists diagnosing images without AI support by disease were as follows: for infectious keratitis, bacterial keratitis had an accuracy rate in the first test of 53.9 ± 27.7%, fungal keratitis had 87.2 ± 8.3%, and acanthamoeba keratitis had 81.2 ± 8.0%. For immunological keratitis, the accuracy rates in the first test were 72.9 ± 27.8% for peripheral ulcerative keratitis, 50.0 ± 33.5% for marginal keratitis, and 55.3 ± 37.5% for phyctenular keratitis. Table 2 represents the changes in accuracy rates for each group of ophthalmologists when assisted by correct CorneAI support and when referencing misleading AI outputs in the second test. In the analysis by disease, corneal specialists exhibited no significant difference in accuracy rates when referencing misleading AI outputs. In contrast, board-certified specialists showed a significant decrease in accuracy only for peripheral ulcerative keratitis when referencing misleading AI outputs. Among residents showed significant improvement with CorneAI support for fungal keratitis and acanthamoeba keratitis, but a significant decrease in accuracy for fungal keratitis and marginal keratitis when referencing misleading AI outputs.

**Table 2:** Accuracy rates of each disease and by ophthalmologists, with and without CorneAI and misleading AI outputs.

	All doctors (*N* = 23)					*P* value
	Without CorneAI (7 images)	With CorneAI (7 images)	*P* value	Without misleading AI outputs (3 images)	With misleading AI outputs (3 images)	
Bacterial keratitis (%±SD)	79.5 ± 33.6	90.7 ± 17.1	0.17	27.5 ± 20.5	15.9 ± 20.5	**0.015**
Fungal keratitis (%±SD)	95.0 ± 6.8	97.5 ± 2.3	0.46	81.2 ± 22.3	15.9 ± 35.1	**0.028**
Acanthamoeba keratitis (%±SD)	83.2 ± 18.3	95.0 ± 5.3	0.056	78.3 ± 23.0	66.7 ± 28.9	0.094
Peripheral ulcerative keratitis (%±SD)	95.0 ± 9.5	98.1 ± 3.4	0.28	73.9 ± 13.0	47.8 ± 11.5	**0.0091**
Marginal keratitis (%±SD)	67.1 ± 25.9	81.4 ± 18.9	**0.029**	44.9 ± 10.9	18.8 ± 10.0	0.15
Phyctenular keratitis (%±SD)	80.1 ± 16.0	90.0 ± 17.5	0.51	31.9 ± 24.7	20.3 ± 2.5	0.51

	Corneal specialists (*N* = 7)					*P* value
	Without CorneAI (7 images)	With CorneAI (7 images)	*P* value	Without misleading AI outputs (3 images)	With misleading AI outputs (3 images)	
Bacterial keratitis (%±SD)	87.8 ± 22.5	95.9 ± 7.0	0.23	28.6 ± 37.8	23.8 ± 41.2	0.42
Fungal keratitis (%±SD)	100	100	N/A	81.0 ± 21.8	57.1 ± 37.8	0.13
Acanthamoeba keratitis (%±SD)	89.8 ± 15.9	93.9 ± 11.2	0.17	95.2 ± 8.3	90.5 ± 8.3	0.42
Peripheral ulcerative keratitis (%±SD)	98.0 ± 5.3	100	0.36	90.5 ± 8.2	71.4 ± 5.2	0.057
Marginal keratitis (%±SD)	79.6 ± 21.836.8	85.7 ± 21.8	0.36	33.3 ± 8.2	33.3 ± 16.5	0.99
Phyctenular keratitis (%±SD)	89.8 ± 15.9	100	0.14	42.9 ± 4.9	33.3 ± 29.7	0.63

	Board-certified ophthalmologists (non-corneal specialists) (*N* = 7)					*P* value
	Without CorneAI (7 images)	With CorneAI (7 images)	*P* value	Without misleading AI outputs (3 images)	With misleading AI outputs (3 images)	
Bacterial keratitis (%±SD)	81.6 ± 33.7	91.8 ± 16.2	0.18	28.6 ± 24.7	9.5 ± 8.2	0.18
Fungal keratitis (%±SD)	83.7 ± 22.5	100	0.10	76.1 ± 29.7	52.4 ± 43.6	0.20
Acanthamoeba keratitis (%±SD)	83.7 ± 20.9	98.0 ± 5.4	0.11	76.2 ± 29.7	57.1 ± 49.5	0.27
Peripheral ulcerative keratitis (%±SD)	94.9 ± 10.8	95.9 ± 7.0	0.99	76.2 ± 8.4	38.1 ± 8.1	**0.015**
Marginal keratitis (%±SD)	57.1 ± 21.8	81.6 ± 25.7	0.061	47.6 ± 21.8	9.5 ± 16.5	0.21
Phyctenular keratitis (%±SD)	79.6 ± 13.9	85.7 ± 27.4	0.59	33.3 ± 29.7	14.3 ± 0	0.38

	Residents (*N* = 9)					*P* value
	Without CorneAI (7 images)	With CorneAI (7 images)	*P* value	Without misleading AI outputs (3 images)	With misleading AI outputs (3 images)	
Bacterial keratitis (%±SD)	71.4 ± 45.3	85.7 ± 26.2	0.18	25.9 ± 17.0	14.8 ± 16.9	0.22
Fungal keratitis (%±SD)	93.7 ± 5.9	100	**0.030**	85.2 ± 25.7	66.7 ± 29.4	**0.038**
Acanthamoeba keratitis (%±SD)	77.8 ± 21.3	93.7 ± 5.9	**0.036**	66.7 ± 33.3	55.6 ± 33.3	0.21
Peripheral ulcerative keratitis (%±SD)	92.1 ± 16.6	93.4 ± 4.2	0.23	59.3 ± 23.1	37.0 ± 25.7	0.18
Marginal keratitis (%±SD)	65.1 ± 33.0	77.8 ± 19.2	0.14	51.9 ± 17.0	14.8 ± 12.8	**0.038**
Phyctenular keratitis (%±SD)	73.0 ± 23.9	85.7 ± 24.6	0.19	29.6 ± 17.0	7.4 ± 6.4	0.18

Corneal specialists also hold board-certified as ophthalmologists.

Significant values are in bold.

## Discussion

This study revealed that when AI presented incorrect diagnoses, the accuracy of non-corneal specialist ophthalmologists and residents decreased, while the accuracy of corneal specialists did not decline. The field of AI diagnostics is advancing rapidly evolving, with new and increasingly advanced AI systems being developed daily, bringing revolutions to various real-world fields, not only ophthalmology^6,8,19,20^. In medical imaging, AI has demonstrated its capabilities in detecting lung cancer in traditional X-rays and CT scans, as well as in identifying breast cancer through mammography^21,22^. In ophthalmology, AI has been successfully applied for diagnosing glaucoma and diabetic retinopathy^23–25^, and AI-equipped fundus cameras have received approval from the Food and Drug Administration^26^. While these reports confirm AI’s efficacy in the medical field, they also acknowledge that AI does not achieve 100% accuracy. Our study suggests that even if AI has an accuracy rate of around 70%, AI support does not significantly alter the diagnostic performance of ophthalmologists.

Differentiating between infectious keratitis and immunological keratitis is critical in corneal treatment, as it directly influences the decision to prescribe topical steroids. The incidence of these conditions varies by region, and ophthalmologists may sometimes lack sufficient experience, potentially leading them to rely on advanced AI systems^4,10,27^. As we predicted, board-certified specialists and residents were misled by the misleading AI outputs, resulting in a significant decrease in their accuracy rates. In contrast, corneal specialists were not misled by the misleading AI outputs, suggesting that corneal specialists possess a higher level of expertise and critical thinking skills, enabling them to better recognize and disregard incorrect AI suggestions.

With CorneAI support, the accuracy of ophthalmologists increased for all images, consistent with previous reports^14^. However, with the support of misleading AI outputs, the accuracy of ophthalmologists decreased for all images. Notably, the accuracy for bacterial keratitis was particularly low when supported by misleading AI outputs. Given that bacterial keratitis can lead to severe visual impairment and, eventually, to blindness^28,29^.

Additionally, board-certified specialists and residents demonstrated lower accuracy rates for both infectious keratitis and immunological keratitis, specifically marginal keratitis and phyctenular keratitis, compared to corneal specialists. The accuracy rates for diagnosing marginal keratitis and phyctenular keratitis among board-certified specialists and residents were particularly low, often falling below 20%. Peripheral ulcerative keratitis is known for its distinct diagnostic features^30,31^, whereas marginal keratitis and phyctenular keratitis fewer characteristic findings, making diagnosis more challenging^32,33^. Consequently, board-certified specialists and residents, who do not specialize in the cornea disease, likely struggled with these diagnoses and were misled by the misleading AI outputs. In summary, accurate diagnostic support from CorneAI for infectious keratitis potentially resulting in corneal blindness and diagnostically challenging immunological keratitis has the potential to reduce misdiagnoses by ophthalmologists and contribute to blindness prevention.

This study revealed that corneal specialists’ accuracy rates did not decline by CorneAI’s misleading outputs. This finding underscores that corneal specialists were not misled by the incorrect AI suggestions due to their superior diagnostic capabilities. Despite the AI’s high error rate in these selected cases, specialists were less likely to be misled by the incorrect AI suggestions, reflecting their deeper clinical experience and diagnostic acumen in the field of corneal diseases. In the future, it is expected that AI support will become prevalent not only in ophthalmology but also in other medical fields^34^. The collaboration between AI and clinicians enhances early diagnostic accuracy, ultimately improving patient outcomes^23^. However, AI does not always produce correct answers. In cases with atypical findings or complex presentations involving multiple conditions, AI may fail to make an appropriate diagnosis^35–37^. Therefore, AI should be considered as an adjunct, with the final diagnosis made by the physician^36,37^. Considering the possibility of AI making incorrect diagnoses, it is essential for medical professionals to acquire specialized knowledge and conduct healthcare accordingly.

## Limitation

There was a significant age difference between corneal specialists and residents. Given that corneal specialists typically possess more years of experience, this result is to be expected. The substantial disparity in accuracy rates between “without CorneAI images” and “without misleading AI outputs” can be attributed to the authors’ deliberate selection of cases that posed a diagnostic challenge between infectious keratitis and immunological keratitis. We should also consider creating misleading AI outputs randomly rather than selectively. However, in real clinical practice, there are many cases where it is difficult to determine whether the condition is infectious keratitis or immunological keratitis. Therefore, this study, which included challenging cases in the misleading AI outputs, is considered to have high relevance to real-world scenarios.

Regarding the proportion of misleading AI outputs, it is unclear whether the 70% correct and 30% incorrect ratio is appropriate. In a preliminary study, two authors (H.M. and Y.U.) experimented a 50% correct and 50% incorrect ratio of AI-generated images. A large number of clearly incorrect AI images led respondents (H.M and Y.U) to distrust the AI’s reliability altogether. As a result, we decided to create the misleading AI outputs with a 30% incorrect ratio instead of 50%.

In this study, the determination of whether the condition was infectious or non-infectious was based exclusively on slit lamp images. This may differ from the outcomes of using CorneAI in real-world scenarios. However, in a future where CorneAI is available, it would likely be used alongside the patient’s medical history, potentially enabling more accurate diagnoses.

## Conclusion

This study demonstrates the potential benefits and limitations of AI-supported diagnosis in ophthalmology, particularly for corneal diseases. While medical AI systems like CorneAI can enhance diagnostic accuracy, their excessive dependance on AI introduces the risk of critical misdiagnoses, especially when faced with rare or challenging cases. Even in a future era coexisting with AI systems, the experience of individual physicians will remain necessary to appropriately interpret AI diagnostic results, and the presence of specialists will continue to be important.

## Electronic supplementary material

Below is the link to the electronic supplementary material.

Supplementary Material 1

Supplementary Material 2

## Data Availability

No datasets were generated or analysed during the current study.
