# Supplementary figures and images for: The importance of clinical experience in AI-assisted corneal diagnosis: verification using intentional AI misleading
Source: Sci Rep. 2025 Jan 9;15:1462. doi: 10.1038/s41598-025-85827-0 (PMC11717947; doi:10.1038/s41598-025-85827-0)

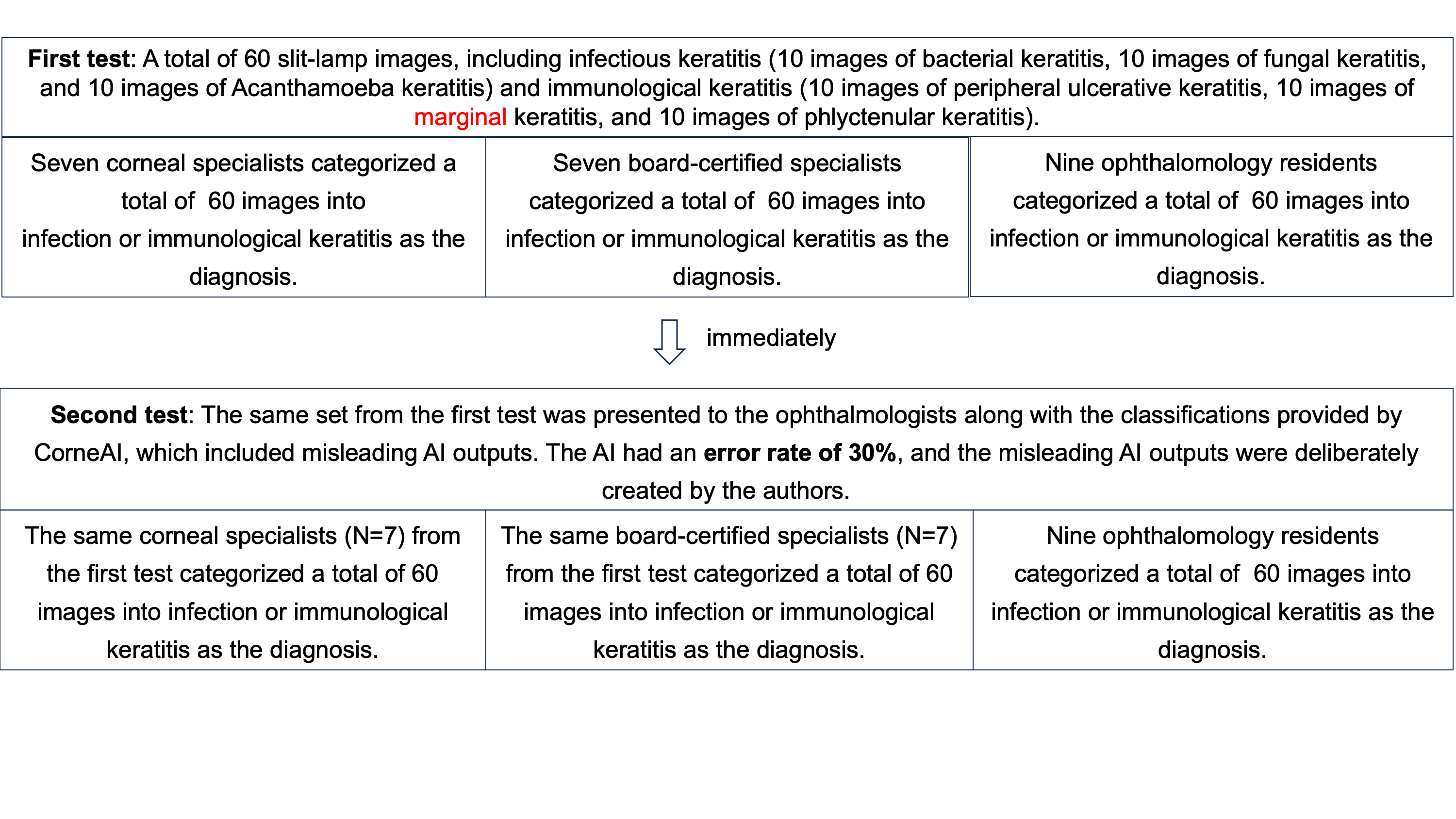

Supplement: Supplementary file 1 — Supplementary Material 1 [file 41598_2025_85827_MOESM1_ESM.tiff]
