# Supplementary material for: The importance of clinical experience in AI-assisted corneal diagnosis: verification using intentional AI misleading
Source: Sci Rep. 2025 Jan 9;15:1462. doi: 10.1038/s41598-025-85827-0 (PMC11717947; doi:10.1038/s41598-025-85827-0)
Supplement: Supplementary file 2 — Supplementary Material 2 [file 41598_2025_85827_MOESM2_ESM.docx]

Fig. S1. **This study’s scheme.**

First, the ophthalmologists were asked to determine whether each of the 60 original images depicted infectious keratitis or immunological keratitis. Subsequently, the same images, including misleading AI outputs, were presented to the ophthalmologists along with the interpretation results from CorneAI for their assessment. For this study, the authors processed the diagnostic results of CorneAI, modifying the images to achieve a correct classification rate of 70% for both infectious keratitis (bacterial keratitis, fungal keratitis, and Acanthamoeba keratitis) and immunological keratitis (peripheral ulcerative keratitis, marginal keratitis, and phyctenular keratitis), with an incorrect rate of 30% for each disease (Misleading AI outputs)
